# Microrefugia and species persistence in the Galápagos highlands: a 26,000-year paleoecological perspective

**DOI:** 10.3389/fgene.2013.00269

**Published:** 2013-12-03

**Authors:** Aaron F. Collins, Mark B. Bush, Julian P. Sachs

**Affiliations:** ^1^Department of Biological Sciences, Florida Institute of TechnologyMelbourne, FL, USA; ^2^School of Oceanography, University of WashingtonSeattle, WA, USA

**Keywords:** Galápagos, fossil pollen, drought, last glacial maximum, extinction, microrefugia, garúa, precipitation

## Abstract

The Galápagos Islands are known to have experienced significant drought during the Quaternary. The loss of mesophytic upland habitats has been suggested to underlie the relatively lower endemism of upland compared with lowland plant assemblages. A fossil pollen record spanning the last 26,000 years from an upland bog on Santa Cruz Island, revealed the persistent presence of highland pollen and spore types during the last glacial maximum and a millennial-scale series of droughts in the mid Holocene. The absence of lowland taxa and presence of mesic taxa led to the conclusion that the highland flora of the Galápagos persisted during both these periods. The resiliency of the highland flora of the Galápagos to long-term drought contradicts an earlier hypothesis that an extinction of highland taxa occurred during the last glacial maximum and that rapid Holocene speciation created the modern plant assemblage within the last 10,000 years. Based on the palynological data, we suggest that, even during the height of glacial and Holocene droughts, cool sea-surface temperatures and strong trade-wind activity would have promoted persistent ground level cloudiness that provided the necessary moisture inputs to maintain microrefugia for mesophytic plants. Although moist conditions were maintained, the lack of precipitation caused the loss of open water habitat during such events, and accounts for the known extinctions of species such as *Azolla* sp., and *Elatine* sp., while other moisture dependent taxa, i.e., *Cyathea weatherbyana*, persisted.

## Introduction

The Galápagos has been an important laboratory for studies in population genetics, extinction, and speciation rates. The majority of these studies focused on adaptive radiation within the islands, the movement of species and incipient species between islands (e.g., Darwin, 1845; Grant and Grant, 1981, 2002; Grant et al., 2000; Caccone et al., 2002; Arbogast et al., 2006). Extinctions are harder to study, but several species of vertebrate have been shown to have gone extinct or, at the least, been extirpated from given islands following human contact (Steadman et al., 1991). Human impacts are also suggested to have caused extirpation (defined as loss from an island) of an upland species of *Acalypha* from San Cristobal Island in the last century (Restrepo et al., 2012). A study of macrofossils in a bog in the uplands of Santa Cruz Island revealed the apparent loss of the waterwort, *Elatine*, within the last millennium (Coffey et al., 2012). This extinction may have been due to hydrologic changes in the wetlands, or ecological cascades related to altered use of the area by giant tortoises or introduced grazers (Coffey et al., 2012). The only example of an extinction unequivocally attributed to climate change is the loss of an aquatic water fern from San Cristobal Island (Schofield and Colinvaux, 1969). Fossil palynological records from El Junco Crater Lake on San Cristobal revealed that during an undated time prior to the last glacial maximum (probably within the last ice age) a species of the water-fern *Azolla* different from any known on the islands today grew in the lake. The disappearance of this population is attributed to ice-age aridity (Schofield and Colinvaux, 1969; Colinvaux and Schofield, 1976a,b).

Under modern conditions, El Niño Southern Oscillation (ENSO) causes the greatest interannual variability in precipitation within the islands. During the El Niño (negative phase of ENSO) rainfall can increase by an order of magnitude, inducing a greening of the landscape, heavy flowering, and increased seed production compared with normal years (Grant and Grant, 1981, 2002; Grant et al., 2000). Contrastingly, during strong La Niña events, rainfall can be close to zero, with corresponding reduction in foliage, flowering, and seedset. Importantly, this pattern is strongest on low-lying islands or on the north slope of islands, but on southerly facing slopes and at elevations above c. 200 m, the lack of rainfall may be partially offset by water intercepted from regular ground-level fog immersion known locally as garúa (Pryet et al., 2012; Trueman et al., 2013).

Populations of animals and plants within the Galápagos have been profoundly influenced by ENSO variability (Grant and Grant, 1981, 2002; Hamann, 1985; Grant et al., 2000). While La Niña events bring annual-scale drought, decadal- or even longer droughts may have shaped floras.

During the last glacial maximum (LGM; defined as 22,000-19,000 year BP), the ITCZ was located south of its modern position (Newell, 1973; Haug et al., 2001; Koutavas and Lynch-Steiglitz, 2004; Leduc et al., 2007; Hodell et al., 2008). The result of this displacement, coupled with weakening of the Atlantic Meridional Overturning Circulation (AMOC) induced oppositely-phased changes in precipitation pattern in each hemisphere. In the northern tropics, cooling of the LGM resulted in relatively cool dry conditions, whereas cool wet conditions prevailed in the southern tropics (Bradbury, 1997; Bush et al., 2011).

The desiccation of the freshwater lakes and bogs within the Galápagos highlands led researchers to conclude that southerly migration of the ITCZ resulted in reduced garúa, prolonged drought and increased aridity within the archipelago during the LGM (Newell, 1973; Colinvaux and Schofield, 1976a,b). There has not been any other terrestrial record that reached the LGM to support or refute the hypothesis of increased aridity with the Galápagos.

Johnson and Raven (1973) postulated that during glacial-aged aridity the xeric-adapted flora of the lowlands expanded upslope replacing the mesic upland flora. If this scenario of the complete extinction of the highland flora and the return of moist conditions to the highlands just c. 10,000 year BP was true, it would set up a phase of rapid speciation to establish the modern upland endemic flora.

The islands are known to have been drier than modern at the last glacial maximum, as lakes dried out and sediments oxidized (Colinvaux and Schofield, 1976a,b). Similarly, between 9000 to 4400 cal. year BP the Intertropical Convergence Zone (ITCZ) migrated northward causing widespread drought in northern and southern hemisphere South America known as the Mid Holocene Dry Event (MHDE) (Gonzàlez et al., 2008; Niemann and Behling, 2008). Within the Galápagos, lake level in El Junco Crater Lake fell and erosion decreased as a consequence of increased aridity during the MHDE (Conroy et al., 2008). Coffey et al. (2012) document the transition of upland pools to bogs during this period on Santa Cruz; consistent with drier, but not arid conditions in the uplands.

Here, we report palynological data from a bog in the Santa Cruz highlands that possesses an intermittent record from 26,200 cal. year BP to 8740 cal. year BP and a complete record from 8740 cal. year BP to modern. We test two competing hypotheses: (1) that the uplands became dominated by xeric vegetation during the last glacial maximum, and (2) that despite overall aridity upland species were able to persist during the glacial maximum.

## Materials and methods

### Site description

**Figure 1:**
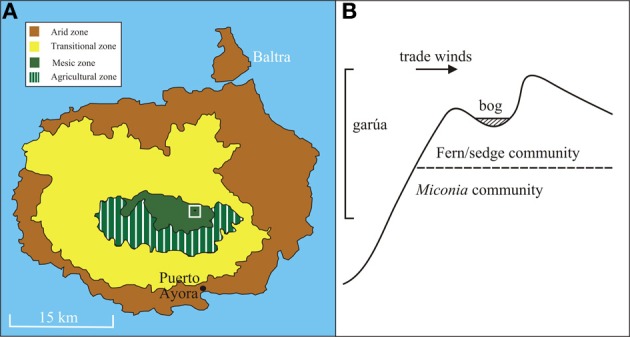
**(A)** Map of Santa Cruz island with associated vegetation zones and agricultural zone within mesic zone highlighted. Paul's Bog (black ellipse) located within white box. **(B)** Diagram of slope of valley with Paul's Bog at the peak with trade wind direction, garúa location and vegetation communities listed.

Paul's Bog, named for Paul Colinvaux, who first cored the bog in 1967 (Colinvaux, 1968), is located in an eroded south-facing cinder cone (0° 38′42.2″ S 90°20′14.4″ W, 800 m elevation) on the island of Santa Cruz with the floor of the bog ~80 m by 40 m (Figure 1). The eroded nature of the cinder cone catchment provides a larger area for pollen rain capture than steep sided basins like El Junco Crater Lake, the only other full Holocene record from the archipelago. The bog lies in the path of prevailing southerly trade winds and is bathed in near constant garúa. The permanent moisture allows modern development of a *Sphagnum* bog, within a Fern/Sedge landscape. The moisture input to the bog provides the possibility of a continuous sediment accumulation during the mid- and late-Holocene.

A diverse bracken fern community is located on the slopes adjacent to the bog, with the area immediately downslope being invaded by the exotic tree *Cinchona pubescens* (Itow, 2003; Jäger et al., 2009). The *Miconia* community (c. 300–550 m elevation) lies just 0.5 km downslope and makes the bog a sensitive location to observe upslope migration of species (Johnson and Raven, 1973) should the garúa have weakened. The shape of the basin limits the potential surface area for past lake formation to c. 0.5 km^2^.

### Core retrieval, processing and sampling

The bog was cored using a 5 cm-diameter Colinvaux-Vohnout piston corer in September, 2004, and again in December, 2005. The longest of the cores raised in 2005 was 627 cm in length. The cores raised in 2004 were split at the University of Washington, while those raised in 2005, were split at the Florida Institute of Technology. Cores were stored at 4°C and imaged using a GEOTEK core-logger of the LUCIE group at the University of Florida. Comparison of the distinctive sediments forming the records of the cores raised in 2004 (400 cm) and 2005 (627 cm) allowed accurate cross-correlation.

Accelerator Mass Spectrometry (AMS) ^14^C radiocarbon dating was conducted on 11 samples (one reversal) from the 2005 core to provide a chronology. Bulk sediment was used (5 cm^3^) for all ^14^C dates because macrofossils were not readily preserved beyond 20 cm depth in the sediment core.

The age model of Paul's Bog was based on ten ^14^C dates from the 2005 core (after removal of age reversal sample) and four bulk AMS ^14^C radiocarbon dates from the core raised in 2004. All dates were calibrated using Calib v6.0 (Stuiver and Reimer, 2005).

Sediment samples of the core raised in 2005 were taken every 2 cm between 7 and 219 cm total depth (*n* = 111). The material from deeper in the core was largely rotted tephra and preliminary surveys showed that pollen was not fossilized. Subsamples were treated with standard chemical protocols to concentrate pollen residues (Faegri and Iversen, 1989; Moore et al., 1991). Each sediment sample was spiked with ~5000 polystyrene microspheres prior to chemical treatment to enable the calculation of pollen concentrations for samples (Battarbee and Kneen, 1982). The top 7 cm of the core was composed of *Sphagnum* moss and was sampled at 1 cm resolution (*n* = 7). Counts were conducted using a Zeiss Axioskop photomicroscope at × 400 and × 1000 magnification, until 250 pollen grains or 2000 microspheres were reached. Pollen identification was based on the modern pollen reference collection of the Florida Institute of Technology. Percentages of taxa and pollen concentrations (grains/cm^3^) within samples were calculated and concentrations were plotted using C2 software (Juggins, 2003).

Samples were ordinated using Detrended Corespondence Analysis (DCA) (Gauch, 1982; Birks, 1985, 1998; Bush, 1991) using PC-ORD v4.41 (McCune and Mefford, 1999). Due to poor pollen preservation in the glacial-aged samples, presence/absence data were used when ordinating glacial-aged and Holocene samples with DCA. All pollen and spore taxa that passed a persistence filter of presence in at least five samples (53 taxa) were used in the analysis. Pollen preservation improved markedly above 120 cm (8740 cal. year BP). A Holocene-only dataset of pollen percentage (0-8740 cal. year BP, *n* = 70), was also ordinated using DCA. The distance measure used was Bray-Curtis dissimilarity coefficient because it reduced the problems of datasets with many zeros (Bray and Curtis, 1957; Minchin, 1987; Pandolfi and Minchin, 1995; Pandolfi and Jackson, 2001). Rare taxa were down weighted.

An ANOSIM analysis was run with PRIMER v5.2.9 (Clarke and Gorley, 2001) on five major groups of Holocene samples located within the DCA to test for a significant difference between the groups (Table 1). The goal of this analysis was to test the DCA outputs for significant changes in community composition before, after, and during the MHDE.

**Table 1:** **Groups of samples for ANOSIM analysis with age range of groups and sample size for each group**.

**Group name**	**Age range (cal. year BP)**	***n***
PB-6	0–1410	7
PB-5	1410–3660	12
PB-4	3660–6600	24
PB-3	6600–7800	20
PB-2	7800–8740	7

Groups were based on clustering by results of a DCA of Holocene-age pollen precentage.

Because pollen data can have many zeros, but can also be dominated by a few taxa in a sample, the data were square root transformed and standardized by the maximum abundance of the dominant taxon in each sample (after Faith et al., 1987). A *post-hoc* permutation test (10,000 replications) was run to detect which pairs of groups significantly differed.

## Results

### Sediment description

**Figure 2:**
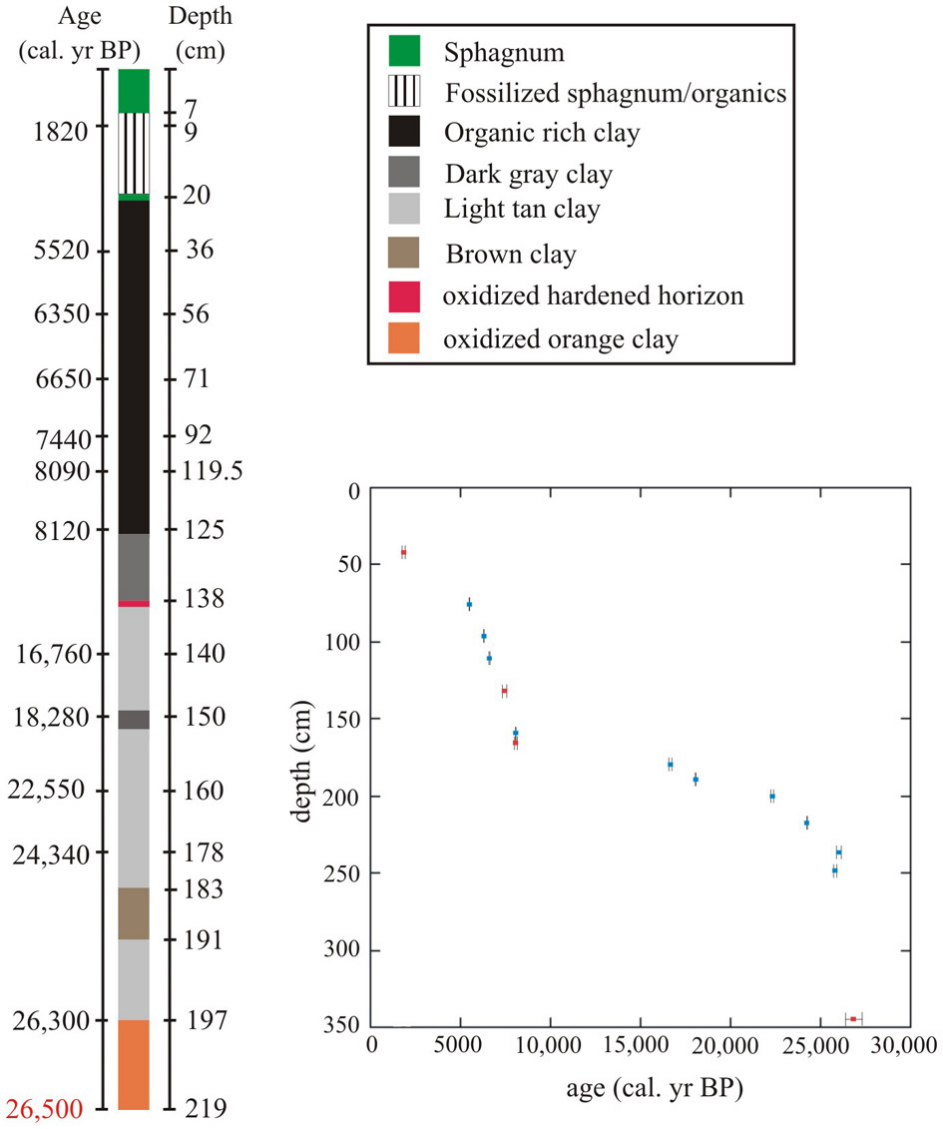
**Core description for 219 cm sampled for pollen analysis (total core length 627 cm)**. Samples subjected to ^14^C analysis have age (left) and depths (right) listed on either side of the core. The 26,500 cal. year BP mark is an interpolated age for the lowest sample taken for fossil pollen analysis, not an actual ^14^C date. Depths for points of change in sedimentology are only marked at right side of core. Inset: Paul's Bog age model for the last 26,000 cal. year BP based on a ^14^C chronology. Red rectangles signify dates that were cross-correlated from the 2004 core, blue rectangles signify dates taken from 2005 core. 2 sigma error of ^14^C dates shown by error bars.

The 219 cm portion of the 2005 core that was used for pollen analysis had distinct changes in sediment color and type (Figure 2). The stratigraphic column was primarily composed of clays below c. 127 cm, and organic material above.

Orange sandy clay indicative of oxidizing conditions was present from the core base (627 cm) to 204 cm (26,050 cal. year BP). The clays differed in color and texture and appeared particularly strongly oxidized below 204 cm (Figure 2). A paleosol may be indicated by a band of weathered material at 138 cm depth and would probably mark a major hiatus in the sedimentation of the site.

After the hardened paleosol, organic-rich clays (c. 139–127 cm) gave way to organic material from 127 cm to c. 20 cm and 7 cm depth, when the organic muds were overlain by a mixture of rotted sphagnum and organic sediment, while the section from 7 to 0 cm was comprised of relatively well-preserved sphagnum. The surface *Sphagnum* layer did not appear to be disturbed as fresh material overlay progressively decayed layers.

The 2004 core possessed consistent sedimentary changes located within the 2005 core that was subjected to pollen analysis. The 2004 core had a longer sphagnum peat layer (50 cm) than the 2005 core (20 cm), but organic layers were similar in length, 120 cm in 2004 core and 127 cm in the 2005 core. Beyond the organic layer, the 2004 core has alternating tan and gray clay layers, with heavily oxidized soils beyond 200 cm. The presence and location of these major sedimentary events within both cores made cross correlation of radiocarbon dates from both cores feasible.

### Age model

The age model for the 2005 core was robust, with only one reversal at c. 25,500 cal. year BP (230 cm). The date at c. 28,000 cal. year BP (209 cm) from the 2005 core was removed because to accept it would have required a greater number of ages to be rejected (Table 2, Figure 2).

**Table 2:** **AMS ^14^C radiocarbon dates from cores extracted in 2004 and 2005**.

**Lab No**.	**Depth (cm)**	**Core used**	**^14^C age ± error (years)**	**Calibrated age range (cal. year BP)**	**Median calibrated age (cal. year BP)**
B4C-PC1 2–2.5 cm	9.0	2004	1870 ± 86	1810–1867	1820
76353	36.0	2005	4770 ± 30	5498–5547	5520
76354	56.0	2005	5570 ± 30	6367–6396	6350
76355	71.0	2005	5830 ± 35	6602–6677	6650
B4C-PC1 0–1 m	92.0	2004	6520 ± 111	7413–7505	7440
76356	119.5	2005	7260 ± 35	8089–8112	8090
B4C-PC2 25–25.5 cm	125.0	2004	7330 ± 74	8041–8186	8120
76357	140.0	2005	13,600 ± 75	16,665–16,868	16,760
76358	150.0	2005	15,150 ± 55	18,481–18,579	18,280
76359	160.0	2005	18,950 ± 80	22,833–22,888	22,550
76360	178.0	2005	20,400 ± 70	24,218–24,485	24,340
76361	197.0	2005	21,900 ± 100	25,933–26,762	26,300
76362	209.0	2005	24,000 ± 160	28,580–29,115	28,850
76363	230.0	2005	21,700 ± 90*	25,865–26,174*	26,030*
B4C-PC4 5	305.0	2004	22,340 ± 466	26,407–27,340	26,880

All samples were bulk organic sediment. Asterisks denoted age reversals.

The lowest meter of the 2005 core subjected to pollen analysis (200–300 cm; c. 26,800-26,300 cal. year BP) had the fastest sedimentation rate (7.6 years/cm) in the entire record. From 200-120 cm (c. 25,310-8100 cal. year BP) the 2005 core had the slowest sedimentation rate in the record (100–400 years/cm). The top 120 cm of the 2005 core (c. 8100-0 cal. year BP) had a moderate sedimentation rate (20–40 years/cm) that was less variable than the previous 80 cm. The sampling resolution was fairly constant (80 years) between 6000 BP and 1400 BP, but the faster sedimentation produced a sampling interval of c. 30–40 years, between 6000 and 8000 cal. year BP. However, the last 1400 years is poorly represented, as the sedimentation rate was severely reduced (155 years/cm) due to hydrach succession, as a more rapidly sedimenting lake formed into a slowly sedimenting bog (Figure 2).

### DCA analyses of pollen dataset

**Figure 3:**
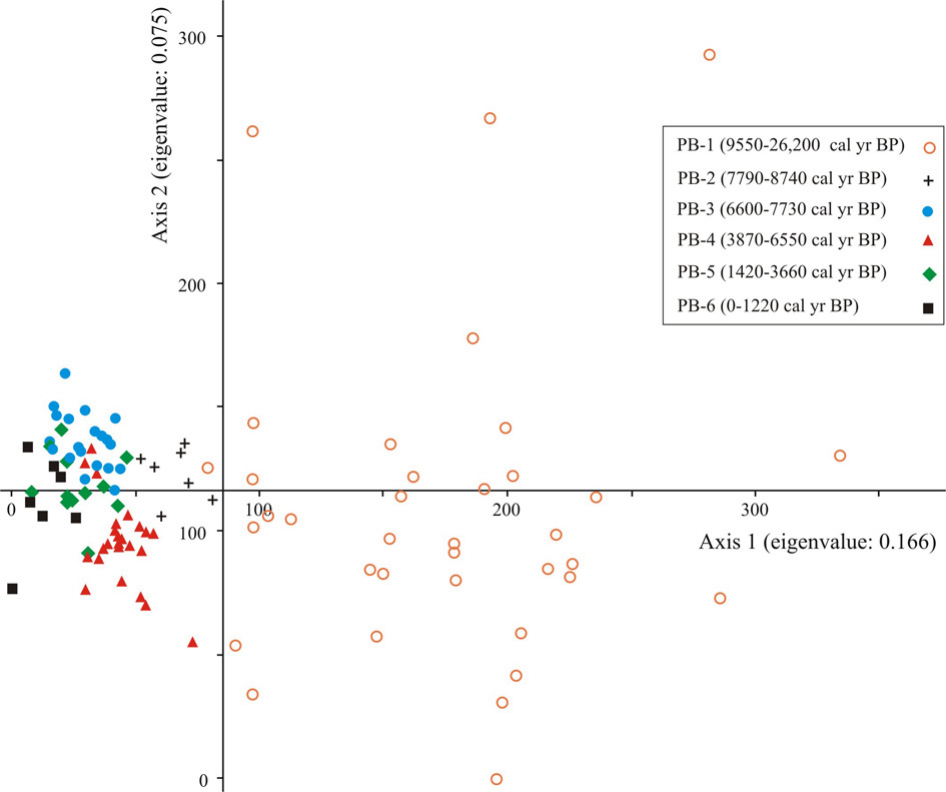
**Ordination results of Axes 1 and 2 of the DCA of presence/absence fossil pollen data for the Paul's Bog dataset (26,200-0 cal. year BP)**. To reduce noise in the dataset, the rarest types were removed. Samples from similar age periods were grouped.

DCA Axis 1 for the entire data set (presence or absence data) separated samples that were older than 8740 cal. year BP from those that were younger. The older samples were characterized by low species diversity and high proportions of Asteraceae (Figure 3).

Pollen taxa that influenced the negative portion of Axis 1 were *Psychotria*, *Myrica*, Solanaceae, and *Acalypha*. Taxa known to be selectively preserved in mildly oxidizing conditions (mixture of Asteraceae types and spores) (Havinga, 1964, 1967, 1984; Moore et al., 1991) influenced the positive extreme of Axis 1.

The negative extreme of Axis 2 was characterized by *Acnistus, Cassia*, and *Ludwigia*. The positive end of the axis had high scores for a mixture of Asteraceae, monolete spores, Myrtaceae, *Podocarpus*, and two unknown types.

**Figure 4:**
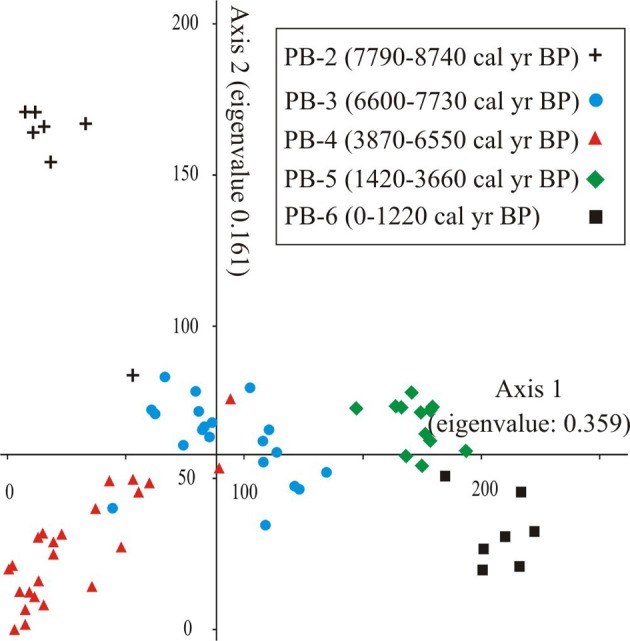
**Ordiantion results of Axes 1 and 2 of the DCA of Holocene-aged pollen samples (8740-0 cal. year BP) from Paul's Bog core**. Groups were labeled by time period.

The DCA of Holocene pollen samples exhibited robust clustering of similarly aged samples in five zones, with relatively few samples that crossed over into the space dominated by samples from another zone (Figure 4). The ordination separated the samples associated with the formation of the lake, PB-2, from those of the MHDE (6600-3660 cal. year BP). Similarly the late Holocene zones were separated from those of the early Holocene.

Marshland components (*Polygonum*, *Ludwigia*, Cyperaceae) characterized the negative extreme of Axis 1 with, *Polygonum* most strongly associated with PB-2 whereas *Ludwigia* dominated PB-3. Most dry land taxa had strong positive scores on Axis 1, e.g., *Zanthoxylum*, *Psychotria*, Myrtaceae, Solanaceae type, Cyperaceae and some lower elevation elements (e.g., *Bursera*, *Cordia*).

### Highland community composition during the LGM, deglaciation and early holocene; local pollen zone PB-1 (26,200-9550 cal. year BP, 128–219 cm)

**Figure 5:**
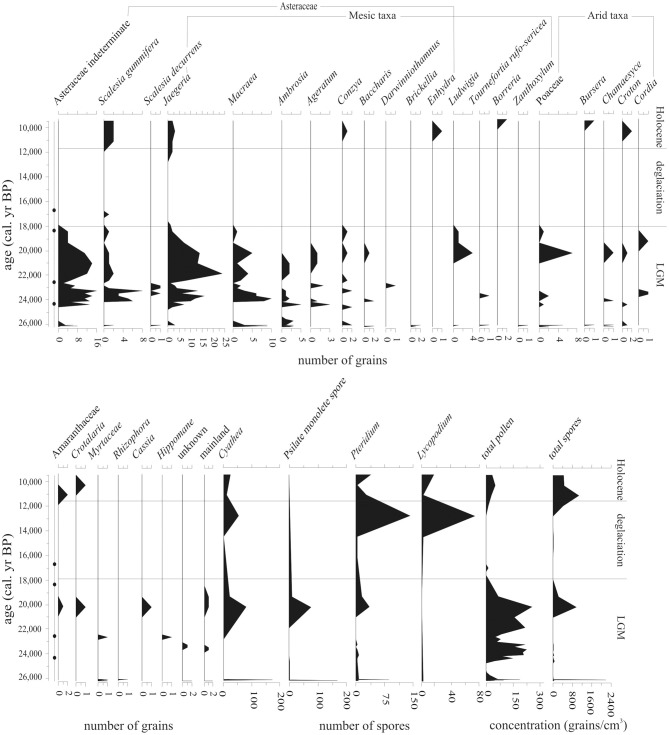
**Pollen and spore abundance (number of grains) of all taxa located within LGM/deglaciation-aged samples (26,200–9550 cal. year BP) from Paul's Bog**. Total pollen and spore concentrations were calculated as grains/cm^3^. Radiocarbon date locations (solid circles) on right of age axis.

Oxidizing conditions cause rapid decomposition of pollen, leaving only palynomorphs with thick exines, e.g., Asteraceae, Poaceae, and spores (Havinga, 1967; Faegri and Iversen, 1989). Despite this selective degradation; enough pollen and spores were preserved to provide qualitative information about conditions around the lake catchment between c. 26,200-9550 cal. year BP (Figure 5).

Spore concentration peaks occurred at c. 26,100 and 20,230 cal. year BP, with a third peak of well preserved spores, albeit at low concentrations, occurring at c. 12,910 cal. year BP. Pollen was preserved in a band of sediment at 26,100 cal. year BP, but was then absent until occurring again between 24,990 and 20,230 cal. year BP. Asteraceae, including highland pollen types associated with modern garúa zones, dominated the pollen preserved in this zone. Concentrations of spores and pollen even in the best of these samples were very low (spores maximum peak: 2180 grains/cm^3^, with 142 grains/cm^3^ sample average; pollen maximum peak: 300 grains/cm^3^, with 67 grains/cm^3^ average) suggesting oxidation and selective preservation.

*Cyathea* spores were present throughout the LGM and early Holocene (c. 26,200-9550 cal. year BP) at levels of 5–30 grains per sample; only one major period (25,500-22,500 cal. year BP) lacked this tree fern. There was a very brief but pronounced (899 grains/cm^3^) peak of *Cyathea* at 26,100 cal. year BP, as well as two peaks of 124 and 399 grains/cm^3^ at 20,230 and 12,910 cal. year BP, respectively.

Unknown psilate monolete spores co-occurred with *Cyathea*, but the 20,230 cal. year BP peak was less marked than for *Cyathea*. Throughout the record, *Pteridium* was rare (4–180 grains/cm^3^), but ubiquitous with only a few samples lacking this taxon. The only substantial peak, 439 grains/cm^3^, occurred at 26,100 cal. year BP.

*Pteridium* spores exhibited peaks at 26,100, 20,230, and 12,910 cal. year BP (385, 381, and 839 grains/cm^3^, respectively), but were present in low concentrations throughout the entire record with 4–98 grains/cm^3^ per sample. *Lycopodium* exhibited a distinct peak of 15 grains at 20,230 cal. year BP, but was absent from most of the record, with concentrations of 5–5.5 grains/cm^3^ when present.

### Highland community composition during the holocene

The results of the DCA conducted on the Holocene data set (8740-0 cal. year BP) grouped similarly aged samples into five clusters (Figure 4). These clusters were used for statistical analyses and pollen zones within the lake basin history of Paul's Bog.

#### Local pollen zone PB-2 (8740-7800 cal. year BP, 118–128 cm)

**Figure 6:**
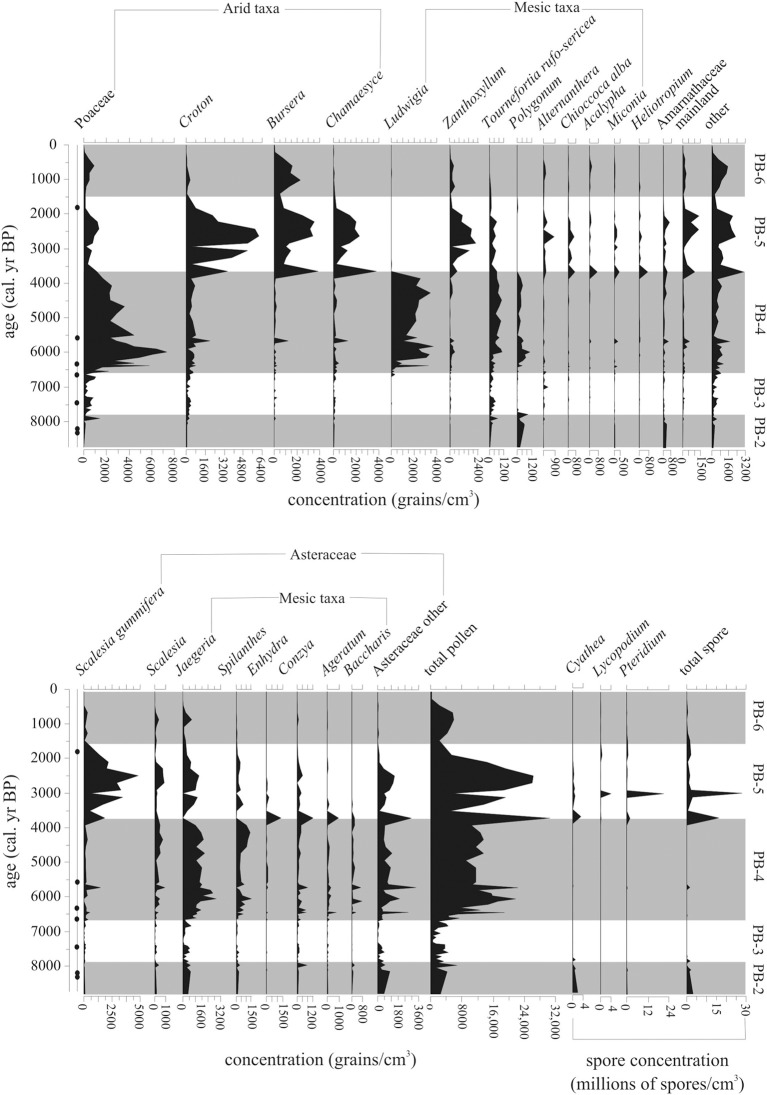
**Pollen concentration (grains/cm^3^) of the 25 most common taxa within the Holocene-aged samples (8740-0 cal. year BP) from Paul's Bog**. Local pollen zones are shown. Radiocarbon date locations (solid circles) on right of age axis.

**Figure 7:**
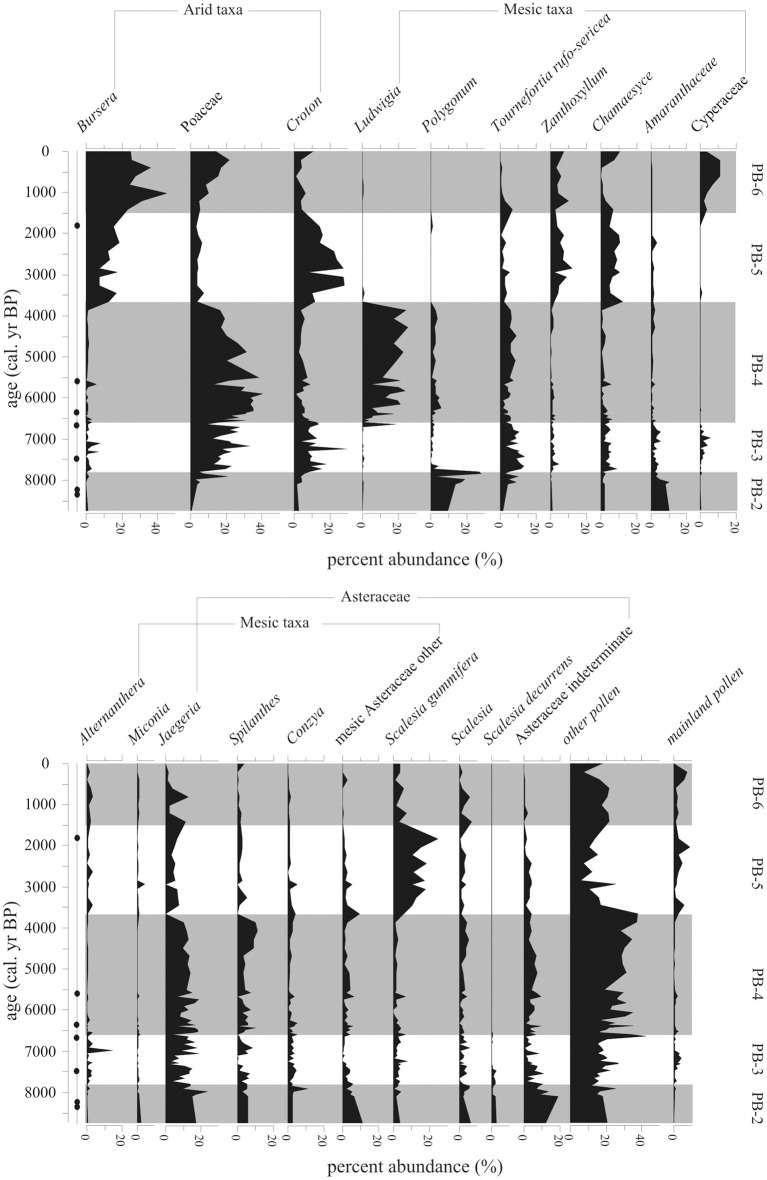
**Percent abundance (%) of 20 most common taxa (>5%) within the Holocene-aged samples (8740-0 cal. year BP) from Paul's Bog**. Local pollen zones are shown. Radiocarbon date locations (solid circles) on right of age axis.

The beginning of this zone had the highest levels (800–1200 grain/cm^3^, 10–20%) of *Polygonum*, an endemic marsh taxon. After 7800 cal. year BP, *Polygonum* fell below 140 grain/cm^3^ (2%) (Figures 6, 7).

Representation of Asteraceae as a family was constant during this zone. Although abundances between the groups varied, the bulk of the Asteraceae came from *Jaegeria* (800–900 grain/cm^3^, 10–18%). *Spilanthes* and *Conzya* were present but remained below 250 grain/cm^3^ (5%) except for a brief 800 grains/cm^3^ (13%) peak of *Conzya* at 7800 cal. year BP. A mixture of other Asteraceae taxa were abundant as a group (c. 1200 grain/cm^3^, 5–10%), but very rare taxon to taxon (c. 50–100 grains/cm^3^, <1%) at the base of the Holocene record. This group of rare Asteraceae taxa began to drop off at 7800 cal. year BP.

Poaceae appeared at 8000 cal. year BP with abundances of 250–500 grains/cm^3^, and a brief peak (c. 1500 grains/cm^3^, 40%) at the termination of zone PB-2. *Cyathea* was extremely numerous (estimated 1,900,000 to 500,000 grains/cm^3^) before 7800 cal. year BP, while other spores were represented at <100,000 grains/cm^3^.

#### Local pollen zone PB-3 (7800-6600 cal. year BP, 79–118 cm)

A strong transition in the pollen flora was evident at 7800 cal. year BP. *Alternanthera* and *Zanthoxylum* occurred after 7800 cal. year BP, but remained below 100 grains/cm^3^ (5%). Cyperaceae was present, but did not exceed 5% during this period. *Alternathera* had one small peak of 450 grains/cm^3^ at 7000 cal. year BP. Amaranthaceae also dropped from 320 grains/cm^3^ to under 100 grains/cm^3^ (10% to 2%) after 7800 cal. year BP.

Pollen concentrations in this zone were the lowest in the record, with the exception of the deglacial (Figure 5) and the last 500 years. The low stand in pollen concentration spanned 7800-6600 cal. year BP. Poaceae, *Polygonum*, *Jaegeria*, and a mix of rare Asteraceae were the main constituents of pollen concentration for this episode. All spores remained very rare during this zone.

#### Local pollen zone PB-4 (6600-3660 cal. year BP, 27–79 cm)

Poaceae dominated this period (3000–4000 grains/cm^3^, 20–40%) and had a peak in pollen concentration of 8000 grains/cm^3^ at 5800 cal. year BP (Figures 6, 7). After the peak, concentrations continued to fall to the upper boundary of this zone.

At 6000 cal. year BP *Ludwigia*, a marsh herb, became extremely dominant in the flora (2500–4000 grains/cm^3^, 10–20%), forming a peak at 4000 cal. year BP followed by a rapid disappearance from the record. *Polygonum* appeared with *Ludwigia* and followed the same trend of increase early in PB-4 to 4000 cal. year BP and reduction in abundance, but it remained less numerous (200–400 grains/cm^3^, <5%) than *Ludwigia*.

Percentages of most highland Asteraceae remained at PB-3 levels with only *Spilanthes* having a small peak at 4250-3750 cal. year BP. *Jaegeria* and *Spilanthes* concentration paralleled those of *Ludwigia*, with a rapid decrease at 4000 cal. year BP.

*Tournefortia rufo-sericea* did not vary in percentage abundance during this episode, but concentrations rose from 6500-5500 cal. year BP (1000 grains/cm^3^) and stabilized at these levels after 3660 cal. year BP.

**Figure 8:**
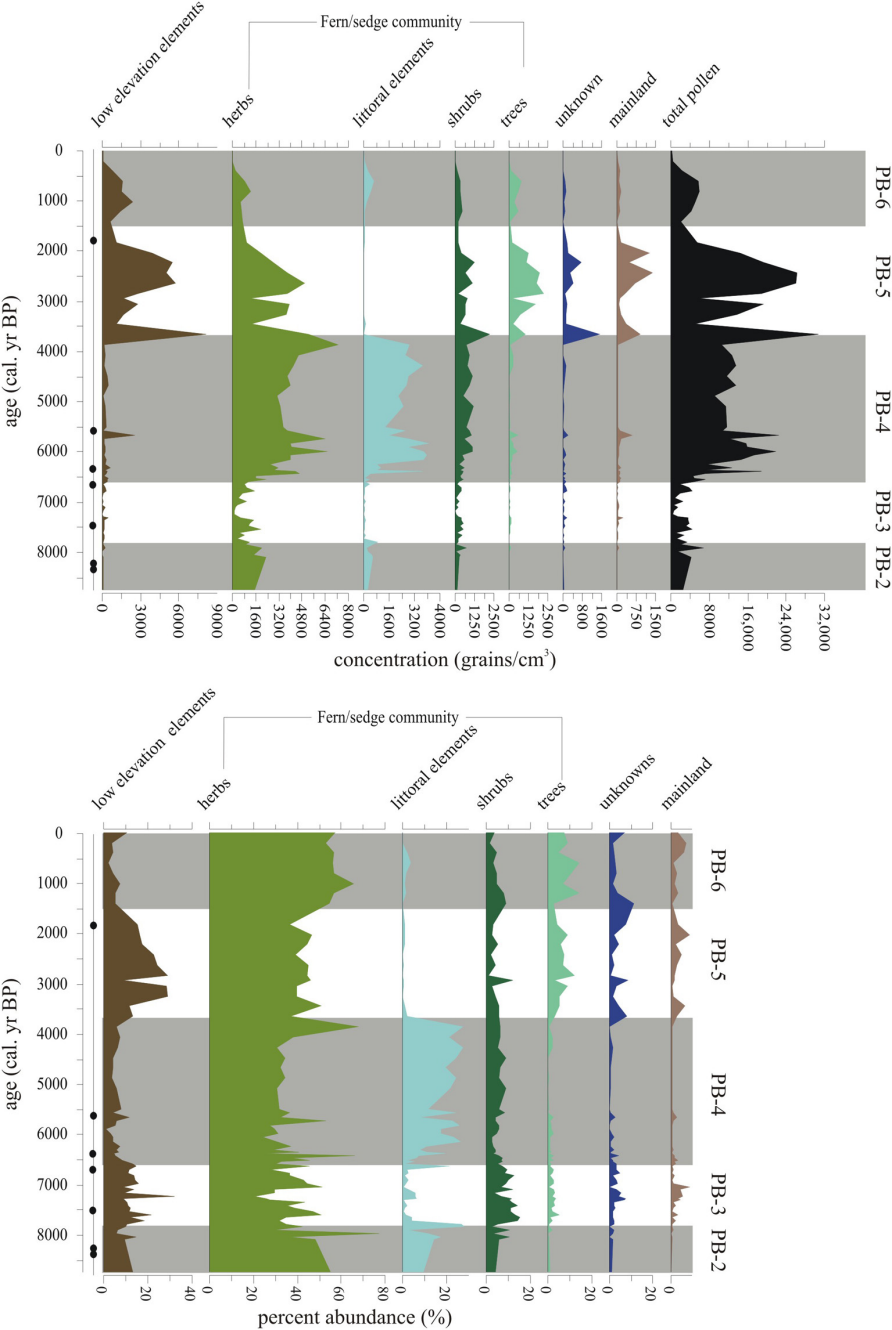
**Summary diagram of the concentrations (grains/cm^3^) and percent abundance (%) of Holocene-aged vegetation types represented in Paul's Bog**. Local pollen zones are shown. Radiocarbon date locations (solid circles) on right of age axis.

Shrub components of the flora (majority *Miconia*) began to show up in the concentration record (400–500 grains/cm^3^), with a plateau similar to *Tournefortia rufo-sericea* (Figures 6–8). Herbs from the Fern/Sedge community generally increased throughout the zone (chiefly mesic Asteraceae, *Tournefortia rufo-serica*, *Acalypha*).

#### Local pollen zone PB-5 (3660-1500 cal. year BP, 7–27 cm)

This episode was characterized by increased representation of taxa from lower elevation zones (Figure 8). *Bursera* percentages increased over the entire period, but concentrations had more variability (1000–4000 grains/cm^3^), with a 1500-year oscillation suggested in PB-5 and PB-6 (Figure 6). *Croton* and *Chamaesyce*, common components of arid and transitional communities, peaked in concentration, 6400 and 4000 grains/cm^3^, respectively, between 3750-2000 cal. year BP. Concentrations of mainland taxa (chiefly *Alnus*, *Podocarpus*, *Myrica*, *Myrsine*), *Croton* and *Chamaesyce* had similar oscillations to *Bursera* (Figure 6). *Scalesia* types (dominant transitional community canopy components) dominated the flora with peaks (3000–6000 grains/cm^3^) in concentration at 3750-1500 cal. year BP.

Poaceae, which had dominated zones PB-3 and PB-4 returned to PB-2 concentrations (c. 2000 grains/cm^3^) during the period of 3660-1500 cal. year BP. *Zanthoxylum* had a concentration high stand (1200–2400 grains/cm^3^) in the core record, while percentages were steady (5–10%) throughout the entire pollen zone.

#### Local pollen zone PB-6 (1500-0 cal. year BP, 1–7 cm)

**Figure 9:**
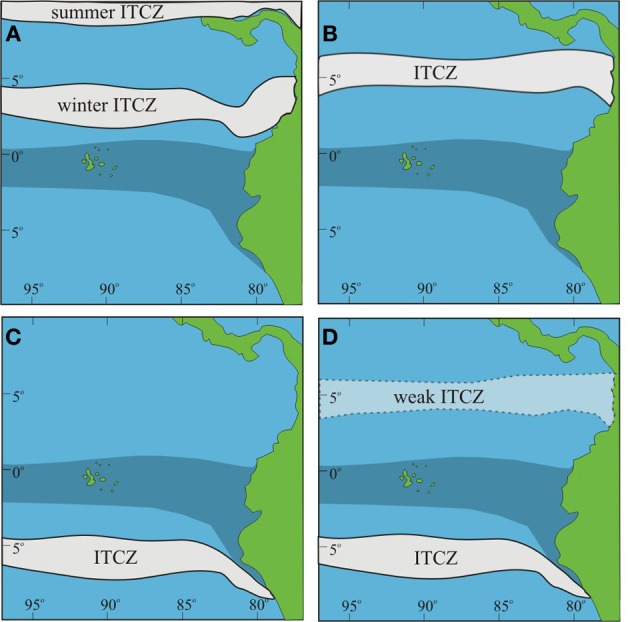
**Scenarios for ITCZ formation during the LGM**. Upwelling off South American coast is shaded. **(A)** Modern ITCZ location; **(B)** ITCZ constrained to northern hemisphere; **(C)** ITCZ constrained to southern hemisphere; **(D)** ITCZ in southern hemisphere with weak ITCZ manifested in northern hemisphere.

Poaceae and *Bursera* increased in abundance in the last 1500 years (Figures 8, 9). Cyperaceae reached their highest percentages in the entire core record. *Zanthoxylum* percentages remained at PB-5 levels (under 500 grains/cm^3^) from 1500 to modern. Taxa concentrations in general were lower during this zone compared with PB-4 and PB-5 with the exception of *Bursera* and *Scalesia* spp., which had maxima within the middle of this zone.

### Anosim analysis of holocene pollen zones

While local pollen zones are routinely assigned to help organize analysis of a fossil pollen record, differences between the zones are seldom quantified. Here, ANOSIM was used to test whether samples in the local zones differed from one another. The zones located within the Holocene pollen diagram (Figure 7) were significance tested with an ANOSIM analysis. A significant difference existed between the groups (*R* statistic 0.843, *p* < 0.001, α = 0.05, *n* = 70), and subsequent *post-hoc* analysis (10,000 permutations) found that every group with the exception of PB-6 and PB-2 significantly differed from each other (Table 3).

**Table 3:** **Pair wise *post-hoc* permutation test (10,000 replications) results for ANOSIM analysis of Holocene local pollen groups described in Table 1**.

**Group comparison**	***R* Statistic**	***p*-value**
PB-6 vs. PB-5	0.729	<0.001
PB-6 vs. PB-4	0.998	<0.001
PB-6 vs. PB-3	0.931	<0.001
PB-6 vs. PB-2	1.00	*P* = 0.1
PB-5 vs. PB-4	0.974	<0.001
PB-5 vs. PB-3	0.797	<0.001
PB-5 vs. PB-2	0.991	<0.001
PB-4 vs. PB-3	0.723	<0.001
PB-4 vs. PB-2	0.864	<0.001
PB-3 vs. PB-2	0.689	<0.001

Importantly, time slices that lay within the period of the MHDE (PB-2, PB-3, and PB-4) were different from each other and from periods accepted as post-MHDE (PB-5 and PB-6).

## Discussion

### Mechanisms of long term drought

There were two prolonged drought phases in the Paul's Bog record. During the deglaciation and early-Holocene (26,000-9000 cal. year BP), Paul's Bog supported an ephemeral pond that only preserved small amounts of pollen and spores. The second phase of aridity occurred while Paul's Bog was a fluctuating shallow lake between 6500-3750 cal. year BP when littoral pollen taxa concentration was at maxima.

#### The full glacial period

During the LGM, the general reduction in sea-surface temperatures in the Pacific Ocean were c. 1–3°C cooler than modern (Koutavas et al., 2002; Lea et al., 2006; Koutavas and Sachs, 2008). Data for the persistence of ENSO during the LGM (Tudhope et al., 2001) suggests that the cool tongue of water that characterize the modern eastern equatorial Pacific (EEP) was probably also present during the LGM (Lea et al., 2002; Stott et al., 2002; Chaing, 2009). Consequently, the temperature differential between the upwelling and the adjacent tropical ocean persisted. The presence of ENSO during the LGM would have allowed for wet and dry oscillations within the Galápagos highlands. The exact primary phase of ENSO variability within the LGM is debated. A persistent El Niño phase has been proffered that would have increased rains to the highlands, but lifted the garúa increasing drought stress on the mesophytic flora of the highlands leading to a possible expansion of arid lowland taxa (Johnson and Raven, 1973; Koutavas et al., 2002; Stott et al., 2002; Rein et al., 2005). An alternate theory is that the EEP cold tongue was accelerated resulting in a persistent La Niña-like state that would have reduced rainfall to the Galápagos highlands, but left garúa in place to reduce evaporative stress in the highlands (Martinez et al., 2003; Lee and Poulsen, 2006).

Pollen preservation requires organic-bearing sediments to be rapidly covered by subsequent sediment layers to prevent oxidation. The periods of mesic pollen preservation within Paul's Bog would be the result of increased rains quickly burying and preserving pollen during mesic periods during a generally dry LGM. The wet phases appear to align to periods of above average LGM sea-surface temperatures (Lea et al., 2006). It is noteworthy that at this location Heinrich Event 2 is a time of warm SST compared to the rest of the LGM and would support a wet episode on the islands due to increased convection (Koutavas et al., 2002; Lea et al., 2006). As the other Heinrich events appear to fall on warm episodes during between 30,000 and 50,000 cal. year BP, it is our working hypothesis that these would have been wet episodes on the islands. Indeed the glacial maximum, if taken as being 20,000 to 22,000 was also wet, consistent with tropical records from Yucatan, Ecuador, and Peru, though apparently different from records from Colombia and Venezuela (Bush et al., 1992; Baker et al., 2001; Hillyer et al., 2009; Correa-Metrio et al., 2010, 2012).

However, most of the previously published records deal with moisture derived from the Atlantic rather than the Pacific. In the Pacific the migration of the ITCZ is complicated by the presence of the cold upwelled waters that inhibit a southward migration during non-El Niño years. The ITCZ cannot form over the upwelling resulting in the ITCZ being constrained to lie in the northern hemisphere (the modern condition). Two possible alternative scenarios under LGM conditions would be: (1) for the ITCZ to form far to the south of its present limit, i.e., south of the upwelling and not migrate past the upwelling (Newell, 1973); or (2) to be split, with a weakened northern range in the boreal summer, an “ITCZ gap” where the upwelling occurs, and a boreal winter location south of the upwelling (sensu Leech et al., 2013) (Figure 9). Leech et al. (2013) suggest that the ITCZ could have transitioned between these various states on a decadal to millennial scale. The presence of wet periods within the LGM EEP supports the hypothesis that the ITCZ was shifting on a millennial scale between a northern and southern hemisphere position. The wet phases increased pollen preservation in the Galápagos highlands as the ITCZ came closer to the islands before bypassing the EEP cold tongue.

Our working hypothesis of the ITCZ occupying, on millennial or sub-millennial timescales, more northern or southern positions than in its current range does not necessarily preclude the alternate hypotheses of a double ITCZ on either side of the equator or a more southerly position within the northern hemisphere during H1 and the LGM (Pahnke et al., 2007; Leduc et al., 2009; Leech et al., 2013).

#### The deglacial period

During the deglaciation, the same slight increases in sea-surface temperature that caused pollen deposition and preservation into Paul's Bog during the full glacial, failed to induce pollen preservation, indicating an overall drier climate after c. 20,000 cal. year BP. The transition toward increasing aridity at this time was also evident in a sudden lowering of lake level at Lake Pacucha in Peru (Hillyer et al., 2009; Valencia et al., 2010).

In general, records from south of the equator register a millennium-long strong dry event c. 20,000 cal. year BP, followed by a wet event that lasted until c. 16,000 cal. year BP (Hillyer et al., 2009; Urrego et al., 2010; Valencia et al., 2010; Mosblech et al., 2012). Records from Panama and the Yucatan, lying at 10° and 17° N, respectively, possess a prolonged dry event until c. 14,000 cal. year BP, an effect of a more southerly mean ITCZ position (Bush and Colinvaux, 1990; Correa-Metrio et al., 2012).

Precessional forcing would have been one of the factors that could have caused the ITCZ to migrate south during the LGM and deglaciation, enhancing rainfall to southern hemisphere lakes and caves (Koutavas et al., 2002; Koutavas and Lynch-Steiglitz, 2004; Cruz et al., 2005; Urrego et al., 2010; Mosblech et al., 2012). Northern Neotropical records generally document the early deglaciation as being dry (Bush and Colinvaux, 1990; Correa-Metrio et al., 2012), with sites in Central America, e.g., Petén Itzá, experiencing a peak of aridity from 17,000-14,000 cal. year BP (Correa-Metrio et al., 2010), while others only started to accumulate sediment after 14,300 cal. year BP, e.g., La Yeguada (Panama) (Bush et al., 1992). Contrastingly, in the southern hemisphere, drying began as early as 16,000 cal. year BP, but was most apparent between 9000 and 4400 cal. year BP (Baker et al., 2001; Hillyer et al., 2009). In the Galápagos the LGM was dry, but had brief mesic periods in phase with the southern hemisphere, c. 26,000 to 20,000 and 13,000 cal. year BP.

Forcing from the Atlantic and Laurentide ice-sheet meltwater impacts, plus the overall height and scale of the ice sheet (CLIMAP, 1981; He et al., 2013) could have accounted for the rapid climatic oscillations seen between 19,000 cal. year BP and c.9000 cal. year BP. Evidence for the migration of the ITCZ in this period is found in the opposed signature of wet and dry events at c. 13°S and 17°N (Baker et al., 2001; Vélez et al., 2006; Hillyer et al., 2009; Valencia et al., 2010; Correa-Metrio et al., 2012). Throughout this period the ITCZ apparently formed either north or south of the Galápagos, but did not lie over the islands.

A failure of the ITCZ to arrive at the islands and reduced SST would reduce atmospheric moisture and wet season precipitation. Garúa is present in the highlands because of temperature inversion of moist air over the cold ocean. As garúa formation is dependent on saturated air, reduced evaporation from the ocean could have caused the garúa to move upslope or to form less often. Reduced inundation with the ground-level cloud of garúa would have increased desiccation and reduced the hydroperiod of Paul's Bog. A water table that oscillated between saturated surface soils and oxidizing conditions would have caused the loss of all but the thickest-walled pollen and spores.

On the Galápagos the duration of background arid conditions from 26,000 to 9000 cal. year BP is unusually long for a site in either hemisphere. The equatorial position of the islands made them relatively sensitive to migrations of the ITCZ, meaning that the Galápagos may reflect a northern hemispheric pattern of aridity at the LGM because the ITCZ was biased to the southern hemisphere, and a southern pattern during the early Holocene.

When seasonality increased due to precessional forcing around 11,000 cal. year BP, the ITCZ was already in its modern northern position (Haug et al., 2001). This was a time of falling lake levels and aridity in the southern Neotropics. With the ITCZ lying to the north of the Galápagos the background dry conditions that began prior to the LGM would have continued. Marine isotope records from the EEP, particularly the Galápagos region, described reduced SSTs until c. 12,000 cal. year BP (Koutavas and Sachs, 2008). Prolonged cooler SSTs during the deglaciation and into the Holocene could have resulted in the extended background dry conditions observed in Paul's Bog.

#### The holocene period

The pollen samples associated with the MHDE stood out statistically from the other Paul's Bog samples. The ANOSIM data supported the interpretation of El Junco Crater Lake data that the MHDE was manifested on the Galápagos, and that it was not a simple, uniform event, but rather a period encompassing many climatic events against an overall backdrop of drier-than-modern conditions (Conroy et al., 2008).

During the MHDE, the ITCZ probably lay near the northern limit of its range, amplifying the Walker circulation and upwelling in the EEP. Between c. 9000 and 4400 cal. year BP, relatively weak ENSO cycles seemed to prevail (Sandweiss et al., 2001; Moy et al., 2002), as the overall state resembled La Niña-like conditions with unusually low SSTs (Haug et al., 2001; Koutavas et al., 2002, 2006). In the lowlands, aridity was amplified by the cool SSTs reducing evaporative moisture available to the archipelago, but in the uplands the intensified garúa, prevented dessication. Nevertheless, without the deluging rains associated with El Niño, water level fell in Paul's Bog as the pond dried down to a marsh between c. 6500 and 3750 cal. year BP.

**Figure 10:**
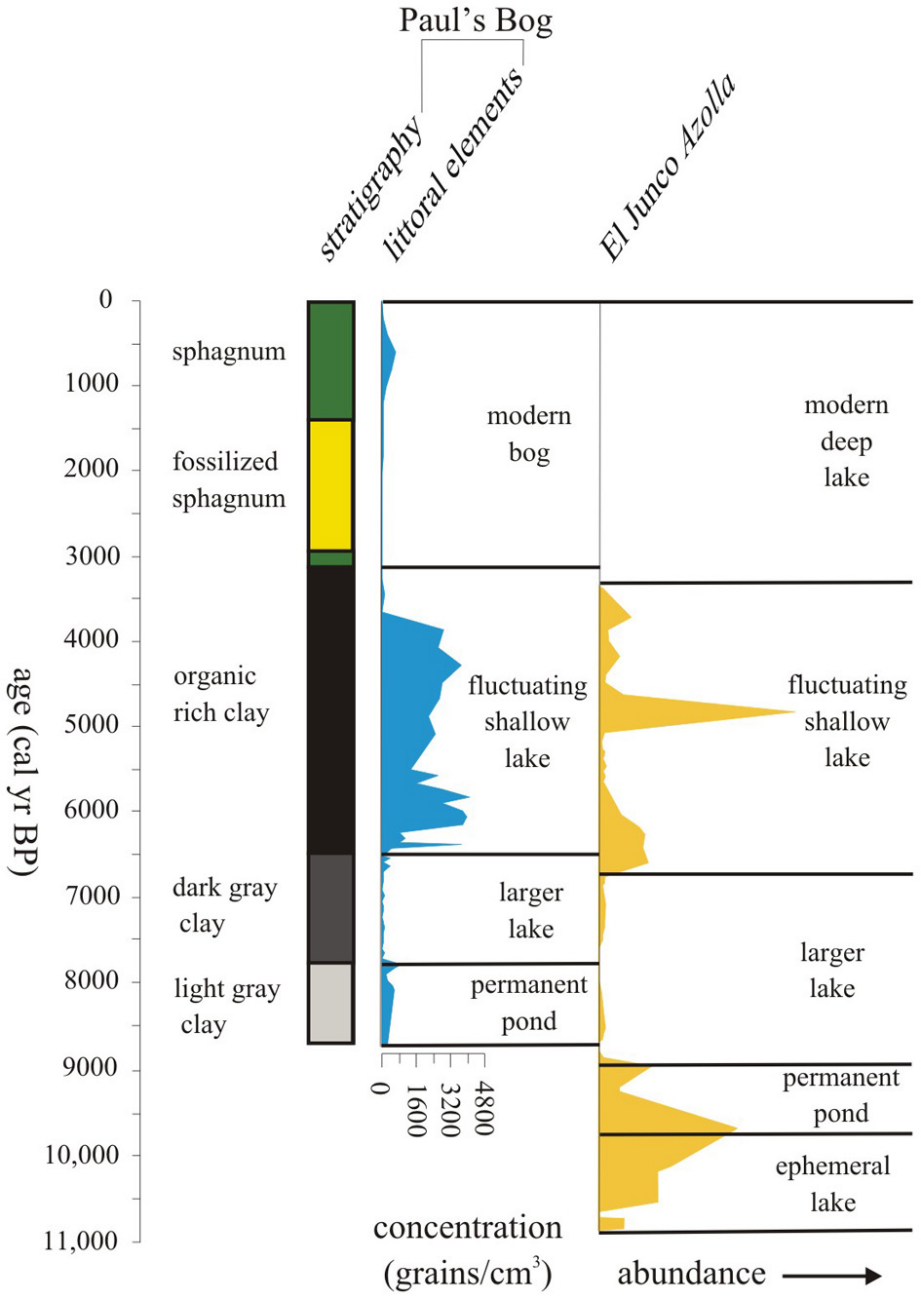
**Lake level history of past 11,000 years using littoral pollen elements and core stratigraphy for Paul's Bog and *Azolla* abundance for El Junco Crater Lake (from Colinvaux, 1972)**.

Although the overall MHDE was manifested on the islands, the local effects of elevation, orientation to the trade winds and thereby, the persistence of local garúa provided slightly different outcomes at Paul's Bog and El Junco Crater Lake (Figure 10). El Junco Crater Lake begins to show a reduction in lake level c. 6750 cal. year BP, while Paul's Bog remained flooded until 6500 cal. year BP (Conroy et al., 2008). An explanation for the differences in time of lake dessication could be the effect of garúa migrating upslope during the MHDE. Paul's Bog lies at 800 m, while El Junco Crater Lake is at 675 m. If the SSTs were lower during the early MHDE, garúa formation could have moved upslope from El Junco Crater Lake, but Paul's Bog could have remained bathed in garúa, reducing evaporation and allowing the basin to become a larger lake early in the MHDE. Also the catchment area for Paul's Bog, is larger proportional to its size than that of El Junco Crater lake allowing greater interception of precipitation.

The MHDE is not believed to be a period of constant aridity along the entire South American mainland, but a drought-prone period between c. 9,000 and 4400 cal. year BP with brief periods of intense rainfall (Valencia, 2006; Vélez et al., 2006; Hillyer et al., 2009). Although the MHDE is generally defined as finishing between 5000 and 4400 cal year BP (Sandweiss et al., 1996; Koutavas et al., 2002; Abbott et al., 2003; Lea et al., 2006), lake levels were continuing to rise in mainland South America until c. 3400 cal. year BP (Bush et al., 2011). Hence, it is not surprising to see this trend toward increased lake levels reflected in the hydrologically sensitive Galápagos wetlands and lakes.

MHDE wet episodes, perhaps the result of intensified El Niño activity or a southern displacement or the ITCZ, were evident on the archipelago c. 8.5 to 6.5 cal. year BP with lake formation at the Paul's Bog catchment. This episode caused desiccation of Colombian swamps and lakes while along the equator an Ecuadorian bog on the eastern flank of the Andes recorded increased precipitation from 8 to 6.5 cal. year BP (Vélez et al., 2006; Gonzàlez et al., 2008; Niemann and Behling, 2008). The Galápagos Islands were dependent on ITCZ-derived precipitation, so a southern shift in the ITCZ during the MHDE would have provided increased moisture. An alternative possibility is that during the peak warming of the Holocene the nature of El Niño events changed, and became more similar to modern Modoki events that have a strong central pacific signature, but induce little change in precipitation in the EEP (Ashok and Yamagata, 2009; Yeh et al., 2009). Thus, rather than looking for a strictly north-south movement of the ITCZ during the Holocene, there may also have been strong east-west gradients in moisture within the Pacific Basin that varied through time as suggested by ocean-atmosphere climate models of the EEP (Leduc et al., 2009).

### Megadroughts and the highland flora

Despite prolonged droughts during the deglaciation and late- to mid-Holocene, the Galápagos does not appear to have undergone wholesale habitat replacement.

Paul's Bog provides information, albeit discontinuous, about the flora of the island back to 26,200 cal. year BP when soils were too oxidized to retain even strong-walled pollen or spores. The oldest palynomorphs retained within the sediment were highland Asteraceae types and spores, including *Cyathea weatherbyana*. This tree fern is moisture dependent and has been used to identify mesic periods around the MHDE (Colinvaux and Schofield, 1976a). The presence of *Cyathea* in Paul's Bog throughout the late-LGM and deglaciation lends support to the idea that the highlands were not completely desiccated, and may have supported the wider fern/sedge community throughout.

Oxidized soils from El Junco Crater Lake dating to >48,000 ^14^C year BP were taken as an indicator of dry conditions in the highlands during the LGM (Colinvaux and Schofield, 1976a). The same soil types from Paul's Bog contained spores, indicating that this portion of the highlands was an area possessing some moisture in order to support *Cyathea* and bracken fern (*Pteridium*). The qualitative appearance of highland elements from Asteraceae lent further support that the windward portions of the Santa Cruz highlands retained moisture and have offered at least microrefugial (sensu Rull, 2005) habitats for mesic taxa during the droughts of the LGM.

The qualitative presence of bracken ferns, *Cyathea*, and highland Asteraceae suggests the continued presence of mesic habitats through the late glacial period. For this system to maintain the mesic elements argues against the complete loss of garúa. The exposed windswept location of Paul's Bog and the other pocket wetlands would dry out very quickly without cloud cover. Our data do not invalidate Johnson and Raven's (1973) argument that loss of habitat area reduced the capacity of endemic taxa to evolve and persist in the highlands. However, our data point to the long-term persistence of the montane endemics such as *Cyathea weatherbyana*, rather than a Holocene invasion and recent speciation.

Conservatively, we can hypothesize that the Galápagos highlands during the LGM were drier than modern, but maintained some garúa cover. Importantly, our data do not indicate an increase of any of the lowland pollen types during the LGM. Microrefugia might have been larger than previously thought (Colinvaux, 1972), possibly located along high elevation (800 m) windward facing highlands, e.g., Paul's Bog, where updrafted moisture was concentrated.

During weaker cold SST events, like the MHDE, garúa does not appear to have been greatly altered because both El Junco and Paul's Bog remained, but were reduced in lake level. Peaks of the littoral taxa, including *Ludwigia* and *Polygonum*, defined the periods of low lake level in Paul's Bog, the apparent dessication of some other bogs on Santa Cruz (Coffey et al., 2012), and were broadly coincident with the peak of the water-fern *Azolla* documented by Colinvaux (1972) in El Junco Crater Lake (Figure 10).

The highland flora did not exhibit significant reductions in diversity during the MHDE, and highland Asteraceae, *Tournefortia rufo-serica*, and *Alternanthera* maintained populations. During the MHDE, La Niña-like conditions were hypothesized to dominate the EEP (Koutavas et al., 2002; Koutavas and Lynch-Steiglitz, 2004). La Niña conditions reduce rainfall to the islands, but garúa cover is strengthened (Snell and Rea, 1999). Under these conditions, the montane vegetation, which is immersed in cloud for much of the year, does not experience the same level of evaporative stress as the lowlands. However, during the peak of the hot season (DJF) there may have been cloudless skies and no rain, increasing evaporation and drought stress. We infer that open water gave way to a marsh at Paul's Bog due to a reduction of precipitation during the MHDE.

The presence of garúa, which acts as a barrier to low elevation taxa (Itow, 2003), would have prevented the upslope expansion of arid taxa. Paul's Bog did not exhibit significant increases in low elevation taxa, providing further support for the persistence of garúa during the MHDE and stability of the highland flora, even with reduced rainfall.

### Resiliency, extinction and speciation of the galápagos flora

Johnson and Raven's (1973) theory of extinction and subsequent rapid speciation of highland elements of the Galápagos during the Holocene cannot be supported by Paul's Bog. Highland taxa appear to have been present in high elevation windward facing peaks and valleys during the deglaciation/early-Holocene period. Presence of pollen from mesophilic taxa at the LGM and the limited community compositional change during the MHDE strengthens the argument for resiliency of the mesic portion of the Galápagos flora.

During the LGM, the Galápagos flora had to contend with oscillations in precipitation, present as periods of pollen preservation and complete oxidation. It has been postulated in the South American mainland that these oscillations allowed downslope migrations of alpine plants in glacials and subsequent population fragmentation as interglacials forced these taxa back upslope (Colinvaux, 1998; Rull, 2005; Mosblech et al., 2012). The highland taxa of the Galápagos apparently persevered in refugia within windward slopes (e.g., Paul's Bog), but the downslope migration probably reflected trends in garuá rather than temperature. In the context of the Galápagos, the distances are so small and the probability of new invasion so slight, the explanation of the observed lowland-highland difference in endemism may have more to do with the number of potential islands that could harbor differentiating populations. Grant et al. (2004) have argued that finches did not radiate *in situ* on each island, but have had complex migrations between islands, radiating and then re-uniting with ancestral types in what they have termed a “braided river” of evolution. The same may be true of lowland plant evolutions, where the larger area of lowlands, and many more islands lacking highlands offer greater potential for speciation, than is available to highland counterparts. Studies of plant geneflow between different island habitats would be particularly revealing.

### Highland flora under future climate change predictions

Future climate models for the EEP are not concordant, with some models predicting decreased ENSO activity under increased greenhouse gas concentrations (Collins, 2000; Cane, 2005; Collins and Groups, 2005), while others predict strengthened ENSO activity (Timmermann et al., 1999; IPCC, 2007b). Under modern ENSO garúa is weakened during El Niño events and strengthened during La Niña events (Snell and Rea, 1999). Increased ENSO activity, particularly more frequent or intense El Niño events, could reduce density of garúa, stressing the highland flora. While decreased ENSO activity would maintain the non-ENSO event state of garúa presence in the highlands.

Increased global temperatures are postulated to have resulted in increased EEP SST and precipitation observed in Galápagos lake records and models predict increased precipitation in the Galápagos region (IPCC, 2007a; Conroy et al., 2009). Increased SST, in conjunction with a predicted weakening of the Walker Circulation could cause an El Niño-like condition in the EEP that would reduce garúa formation in the highlands (Vecchi et al., 2006; Vecchi and Soden, 2007; Sachs and Ladd, 2010). Future warming could result in garúa no longer buffering the highland flora to arid conditions for the first time since the LGM. Garúa formation and mechanics, however, are difficult to predict (Sachs and Ladd, 2010; Pryet et al., 2012). Because garúa is a major factor in the presence of a mesic plant community in an oceanic desert, further study into garúa dynamics is warranted to understand how modeled future ENSO activity and global temperatures will impact the highland flora.

## Conclusions

The flora of the Galápagos has withstood long-term drought for much of the last 26,000 years. Overall, glacial conditions were dry, but sea-surface temperature still appears to have been an important determinant of wet and dry episodes, with the times of maximum cooling in the Atlantic perhaps being most likely to be wet in the Galápagos. Throughout the last 26,000 years, the continued presence of garúa allowed microrefugia to support populations of highland species. The species most susceptible of extinction on the Galápagos appear to have been obligate aquatic organisms. While humid conditions in the uplands appear to have been maintained by cloud cover even if the rainfall is negligible, open water bodies were vulnerable to desiccation. The composition of fossil pollen types reflected lake level, but there was no evidence of lowland taxa invading the highlands. The resiliency of mesic communities within the archipelago in the LGM and Holocene was far greater than previously postulated (Schofield and Colinvaux, 1969; Johnson and Raven, 1973; Colinvaux and Schofield, 1976a, b). With increasing global temperatures postulated to bring further aridity to the Galápagos and the continued rise in invasive species, the flora of the Galápagos faces new threats, quite different to those of the past. Locating Quaternary refugia may outline potential areas for intensive preservation to allow the highland community to persist future periods of long-term drought.

### Conflict of interest statement

The authors declare that the research was conducted in the absence of any commercial or financial relationships that could be construed as a potential conflict of interest.
